# Lyme Disease Under-Ascertainment During the COVID-19 Pandemic in the United States: Retrospective Study

**DOI:** 10.2196/56571

**Published:** 2024-09-12

**Authors:** Brie S Jones, Michael E DeWitt, Jennifer J Wenner, John W Sanders

**Affiliations:** 1 Section on Infectious Diseases, Department of Medicine, Wake Forest University School of Medicine, Medical Center Blvd, Winston Salem, NC, 27159, United States, 1 336-422-7771; 2 Department of Biology, Wake Forest University, Winston Salem, NC, United States; 3 Center for the Study of Microbial Ecology and Emerging Diseases, Wake Forest University School of Medicine, Winston Salem, NC, United States

**Keywords:** surveillance, ascertainment, Lyme diseases, vector-borne diseases, vector-borne disease, vector-borne pathogens, public health, Lyme disease, United States, North Carolina, COVID-19, pandemic, hospital, hospitals, clinic-based, surveillance program, geospatial model, spatiotemporal

## Abstract

**Background:**

The COVID-19 pandemic resulted in a massive disruption in access to care and thus passive, hospital- and clinic-based surveillance programs. In 2020, the reported cases of Lyme disease were the lowest both across the United States and North Carolina in recent years. During this period, human contact patterns began to shift with higher rates of greenspace utilization and outdoor activities, putting more people into contact with potential vectors and associated vector-borne diseases. Lyme disease reporting relies on passive surveillance systems, which were likely disrupted by changes in health care–seeking behavior during the pandemic.

**Objective:**

This study aimed to quantify the likely under-ascertainment of cases of Lyme disease during the COVID-19 pandemic in the United States and North Carolina.

**Methods:**

We fitted publicly available, reported Lyme disease cases for both the United States and North Carolina prior to the year 2020 to predict the number of anticipated Lyme disease cases in the absence of the pandemic using a Bayesian modeling approach. We then compared the ratio of reported cases divided by the predicted cases to quantify the number of likely under-ascertained cases. We then fitted geospatial models to further quantify the spatial distribution of the likely under-ascertained cases and characterize spatial dynamics at local scales.

**Results:**

Reported cases of Lyme Disease were lower in 2020 in both the United States and North Carolina than prior years. Our findings suggest that roughly 14,200 cases may have gone undetected given historical trends prior to the pandemic. Furthermore, we estimate that only 40% to 80% of Lyme diseases cases were detected in North Carolina between August 2020 and February 2021, the peak months of the COVID-19 pandemic in both the United States and North Carolina, with prior ascertainment rates returning to normal levels after this period. Our models suggest both strong temporal effects with higher numbers of cases reported in the summer months as well as strong geographic effects.

**Conclusions:**

Ascertainment rates of Lyme disease were highly variable during the pandemic period both at national and subnational scales. Our findings suggest that there may have been a substantial number of unreported Lyme disease cases despite an apparent increase in greenspace utilization. The use of counterfactual modeling using spatial and historical trends can provide insight into the likely numbers of missed cases. Variable ascertainment of cases has implications for passive surveillance programs, especially in the trending of disease morbidity and outbreak detection, suggesting that other methods may be appropriate for outbreak detection during disturbances to these passive surveillance systems.

## Introduction

The COVID-19 pandemic caused a substantial disruption in the daily routines of many Americans. With restrictions on indoor activities and the increased flexibility of working from home, participation in outdoor activities increased over the course of the pandemic, including park attendance (Figure S1 in Multimedia Appendix 1). Outdoor activities such as running, hiking, fishing, biking, and camping increased during the COVID-19 pandemic [1], as well as a general increase in greenspace utilization [2,3]. With this increased exposure to nature, there is also increased exposure to vectors such as ticks, which can carry vector-borne diseases such as *Borrelia burgdorferi* and *Borrelia mayonii*, the causative agents of Lyme disease [4]. With more individuals spending time outdoors, coupled with the expanding geography of Lyme disease [5], the associated increase in exposure to vector-borne diseases would suggest an increase in number of cases of Lyme disease during this time period. Early research suggests that the reported number of Lyme disease cases decreased in 2020 [6], but there remains questions about the degree of the changing ascertainment rate and the dynamics of this ascertainment rate during the pandemic period.

During the peak of the COVID-19 pandemic, many businesses either transitioned to a remote setting or closed entirely. Similarly, many clinics moved to a telehealth platform, thus limiting access to in-person health care [7]. Furthermore, many individuals lost their job and their access to health insurance [8]. With more individuals staying at home and not pursuing health care advice unless their condition became severe, there is potential that milder disease courses would go undiagnosed. Passive surveillance data are often used by public health officials to detect trends in infectious diseases and identify outbreaks [9]. Changing rates of ascertainment could have large impacts on the ability of public health officials to detect outbreaks where relatively consistent ascertainment rates are required [10].

Given these changes in the frequency of outdoor activity participation and frequency of health care–seeking behavior, we investigated the number of reported cases of Lyme disease in the United States and North Carolina throughout the COVID-19 pandemic and compared these modeled estimates to understand the changes in the ascertainment of cases during this period.

## Methods

### Data Sources

Lyme disease cases that meet the case definition by clinical criteria and/or laboratory criteria are reported to local and state health departments by health care providers. Once personal identifiers are removed, these data are reported to the Centers for Disease Control and Prevention (CDC). The most recent case definition, published by the CDC in 2022, characterizes Lyme disease by 1 or more of the following clinical criteria in the absence of another known etiology: erythema migrans, lymphocytic meningitis, cranial neuritis including facial palsies, radiculoneuropathy, encephalomyelitis, recurrent joint swelling in 1 or a few joints, and acute onset of high-grade atrioventricular conduction defects that resolve within days to weeks [11]. Laboratory criteria include the isolation of the causative pathogens *B burgdorferi* or *B mayonii* in culture, detection of the causative pathogens by nucleic acid amplification test assay, detection of *B burgdorferi* antigens by immunohistochemical assay on biopsy, or positive serologic testing for *B burgdorferi* immunoglobulin M or G [11].

Data on reported numbers of Lyme disease cases were extracted from the cases reported each month in the United States by CDC Lyme Disease Surveillance data sources from 2008 to 2020 [12]. Our study also focused on North Carolina, a state in the southeastern United States, where Lyme disease cases are publicly reported regularly, including at the county level. Monthly North Carolina data were extracted from the North Carolina Department of Health and Human Services (NCDHHS) monthly communicable disease reports for the years from 2017 to 2020. As monthly data were no longer reported using similar formats, a plot digitizer was used to extract the monthly Lyme disease cases from the annual reports from 2021 to 2022. Data on the yearly number of cases by North Carolina county were extracted from NCDHHS’s “Disease Data Dashboard” [13]. Population estimates for the entire United States for each year analyzed were retrieved from the United Nations Division of Population country estimates [14]. North Carolina estimates used the estimated population by county for each year of analysis, using data from the North Carolina Office of Management and Budgets [15].

### Ethical Considerations

This study was approved by the Wake Forest University School of Medicine Institutional Review Board (IRB00117564). All authors had permission to use these data.

### Statistical Methods

We used a Bayesian regression framework fitting data prior to 2020 to estimate the under-ascertainment of cases by month and year. Cyclic cubic regression splines with 12 knots representing each month of the year were used to account for the seasonal dynamics of infection during the year. We fitted these models using both Poisson and negative binomial generalized linear models with log link functions, including population estimates as offsets. We included a random effect to account for the changing case definition when modeling the national case data [16]. Models were fitted using the *brms* R package (R Foundation for Statistical Computing) [17] and assessed using Pareto smoothed importance sampling [18]. In each case, weakly informative priors of Student *t* distribution with 3 degrees of freedom, a mean of 0, and a scale of 1046 were used, with the exclusion of a unit normal distribution prior placed on the case definition random effect. Model convergence was assessed using the Gelman-Rubin diagnostic with values of Rhat less than 1.01 and sufficient effective samples in the posterior estimates. Case ascertainment was defined as the difference between the model estimated number of cases and the reported number of cases: *(Estimated number of cases – Actual number of reported cases) / Estimated number of cases)*.

In a geospatial analysis, we fitted generalized regression models for negative binomial distributed data with a log link function, using an offset for population on the yearly number of Lyme disease cases by North Carolina county. To account for spatial autocorrelation among the counties, we included a smoothed term using the centroid of each county, accounting for both the longitude and latitude of the county centroid. A yearly fixed effect was included to account for the secular increase in the reported number of Lyme disease cases. Model fitting was conducted using the *mgcv* R package [19]. Case ascertainment rates were calculated as defined above.

We conducted all analysis in R (version 4.1.3).

## Results

**Figure 1.**
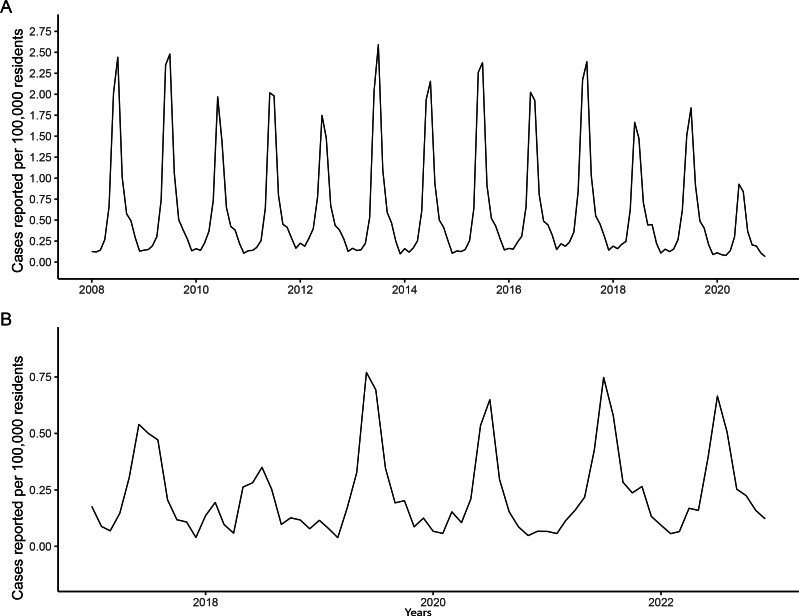
Number of reported Lyme disease cases per 100,000 residents in (A) the United States and (B) North Carolina between 2008 and 2020 by month.

The reported number of Lyme disease cases in 2020 were the lowest both across the United States and North Carolina compared to prior years, with 11,479 (3.42 cases per 100,000 residents versus 6.77 cases per 100,000 residents in 2019) and 264 (2.50 cases per 100,000 residents in 2020 vs 3.29 cases per 100,000 residents in 2019) cases, respectively. These case rates can be seen by month in Figure 1A and B.

All models met the convergence criteria. We found that the negative binomial models best fit the monthly data, and the resulting monthly effects demonstrated a strong seasonal effect, with a higher rate of cases detected during the summer months peaking in July and a nadir occurring between December and February (Figure S2A-D in Multimedia Appendix 1).

Posterior median estimates of the ascertainment rate nationally ranged between 41% to 49% between March and December 2020, the last data point available (Table 1).

This suggests that roughly 14,200 cases may have gone undetected. We estimated that between 40% to 80% of Lyme diseases cases were detected in North Carolina between August 2020 to February 2021, although after this point, ascertainment rates seem to return to normal levels (Table 2).

These months coincide with the peak months of COVID-19 cases in the United States and North Carolina. The spatio-temporal regression model result shows evidence of strong spatial effects (Table S1 in Multimedia Appendix 1).

**Table 1.** Estimated number of reported Lyme disease cases and associated ascertainment rate for the United States from January to December 2020.

Year and month	Estimate, median (95% credible interval)	Ascertainment^a^, median (95% credible interval)
**2020**		
January	488.0 (449.4-532.4)	*0.76 (0.70-0.76)^b^*
February	487.8 (438.0-542.6)	*0.60 (0.54-0.60)*
March	647.2 (581.0-717.9)	*0.41 (0.37-0.41)*
April	986.7 (885.2-1096.6)	*0.46 (0.42-0.46)*
May	2292.3 (2052.3-2564.9)	*0.44 (0.40-0.44)*
June	6564.2 (5900.3-7281.4)	*0.48 (0.43-0.48)*
July	6823.9 (6146.7-7624.3)	*0.41 (0.37-0.41)*
August	2992.8 (2698.7-3317.3)	*0.41 (0.37-0.41)*
September	1685.2 (1513.0-1875.0)	*0.41 (0.37-0.41)*
October	1422.2 (1282.3-1585.2)	*0.45 (0.40-0.45)*
November	884.6 (790.1-985.7)	*0.41 (0.37-0.41)*
December	488.0 (449.4-532.4)	*0.45 (0.41-0.45)*

a Ascertainment represents the proportion of estimated cases that have been reported, defined as *(Estimated – Actual) / Estimated*.

b Italicized values represent where the upper bound of the 95% credible interval does not include 1.

**Table 2.** Estimated number of reported Lyme disease cases and associated ascertainment rate for North Carolina between January 2020 and December 2022.

Year and month	Estimate, median (95% credible interval)	Ascertainment^a^, median (95% credible interval)
**2020**		
January	11.4 (8.6-15.1)	*0.61 (0.46-0.61)^b^*
February	10.6 (7.6-15.1)	*0.57 (0.40-0.57)*
March	9.5 (6.3-14.3)	1.69 (1.12-1.69)
April	14.2 (9.9-20.5)	*0.77 (0.54-0.77)*
May	30.3 (22.5-41.5)	*0.73 (0.53-0.73)*
June	52.2 (39.1-71.0)	1.07 (0.79-1.07)
July	53.4 (39.8-72.3)	1.27 (0.94-1.27)
August	36.0 (26.8-49.0)	*0.86 (0.63-0.86)*
September	20.2 (14.5-28.3)	*0.79 (0.57-0.79)*
October	14.4 (10.5-20.1)	*0.62 (0.45-0.62)*
November	11.9 (8.5-16.4)	*0.42 (0.31-0.42)*
December	11.4 (8.6-15.1)	*0.61 (0.46-0.61)*
**2021**		
January	11.5 (8.7-15.2)	*0.61 (0.46-0.61)*
February	10.6 (7.6-15.2)	*0.56 (0.39-0.56)*
March	9.6 (6.4-14.4)	1.25 (0.83-1.25)
April	14.4 (10.0-20.7)	1.18 (0.82-1.18)
May	30.6 (22.7-41.9)	*0.75 (0.55-0.75)*
June	52.7 (39.5-71.7)	*0.85 (0.63-0.85)*
July	53.9 (40.2-73.0)	1.47 (1.08-1.47)
August	36.3 (27.1-49.5)	1.68 (1.23-1.68)
September	20.4 (14.7-28.5)	1.47 (1.05-1.47)
October	14.6 (10.5-20.3)	1.72 (1.23-1.72)
November	12.0 (8.6-16.5)	2.34 (1.69-2.34)
December	11.5 (8.7-15.2)	1.22 (0.92-1.22)
**2022**		
January	11.6 (8.8-15.4)	*0.86 (0.65-0.86)*
February	10.8 (7.7-15.4)	*0.56 (0.39-0.56)*
March	9.7 (6.4-14.5)	*0.72 (0.48-0.72)*
April	14.5 (10.1-20.9)	1.24 (0.86-1.24)
May	30.9 (22.9-42.3)	*0.55 (0.40-0.55)*
June	53.2 (39.9-72.4)	*0.79 (0.58-0.79)*
July	54.4 (40.6-73.7)	1.30 (0.96-1.30)
August	36.7 (27.4-50.0)	1.47 (1.08-1.47)
September	20.6 (14.8-28.8)	1.31 (0.94-1.31)
October	14.7 (10.7-20.5)	1.63 (1.17-1.63)
November	12.1 (8.6-16.7)	1.41 (1.02-1.41)
December	11.6 (8.8-15.4)	1.12 (0.84-1.12)

a Ascertainment represents the proportion of estimated cases that have been reported, defined as *(Estimated – Actual) / Estimated*.

b Italicized values represent where the upper bound of the 95% credible interval does not include 1.

## Discussion

### Principal Findings

We found evidence of an increase in the under-ascertainment of Lyme disease during the pandemic period, using the historical case rates, in both the United States and North Carolina in 2020. Following February 2021, as COVID-19 cases began to decline in North Carolina (Figure S3 in Multimedia Appendix 1), reported cases of Lyme disease returned to expected levels. This under-ascertainment of Lyme disease demonstrates the importance of a robust surveillance system with well-defined diagnostic criteria of this well-documented emerging infectious disease in the southeastern United States [5,20]. The unique reporting structure of North Carolina allows us to characterize the dynamics of the ascertainment rate at the county level during this period. With the risk of acquiring Lyme disease being the same as years prior, if not higher due to the increased popularity of outdoor recreational activities, it is reasonable to suggest that there was an increased number of cases of Lyme disease that went undiagnosed and untreated during this period. Our estimates should likely be taken as the upper bound of the likely ascertainment rate of Lyme disease cases.

### Engagement With Prior Work

Our findings support early research by McCormick and colleagues [6], who estimated that fewer Lyme disease laboratory tests were reported during the year 2020 than 2019. However, our findings quantitatively establish likely bounds for the numbers of under-ascertained cases of Lyme disease at a national level and at the finer county level in North Carolina, as well as estimate when the normal under-ascertainment of cases resumed. Understanding the dynamics of case ascertainment is vital, especially for passive surveillance systems that are already biased toward persons who seek health care [9,21]. Passive disease surveillance data are often used to feed models to identify potential outbreaks of infectious diseases [10]. Large variations in the ascertainment rates could potentially mask outbreaks of disease or similarly trigger action for case investigations when it is not warranted.

### County Population Differences Between Expected and Reported Lyme Disease Cases

**Figure 2.**
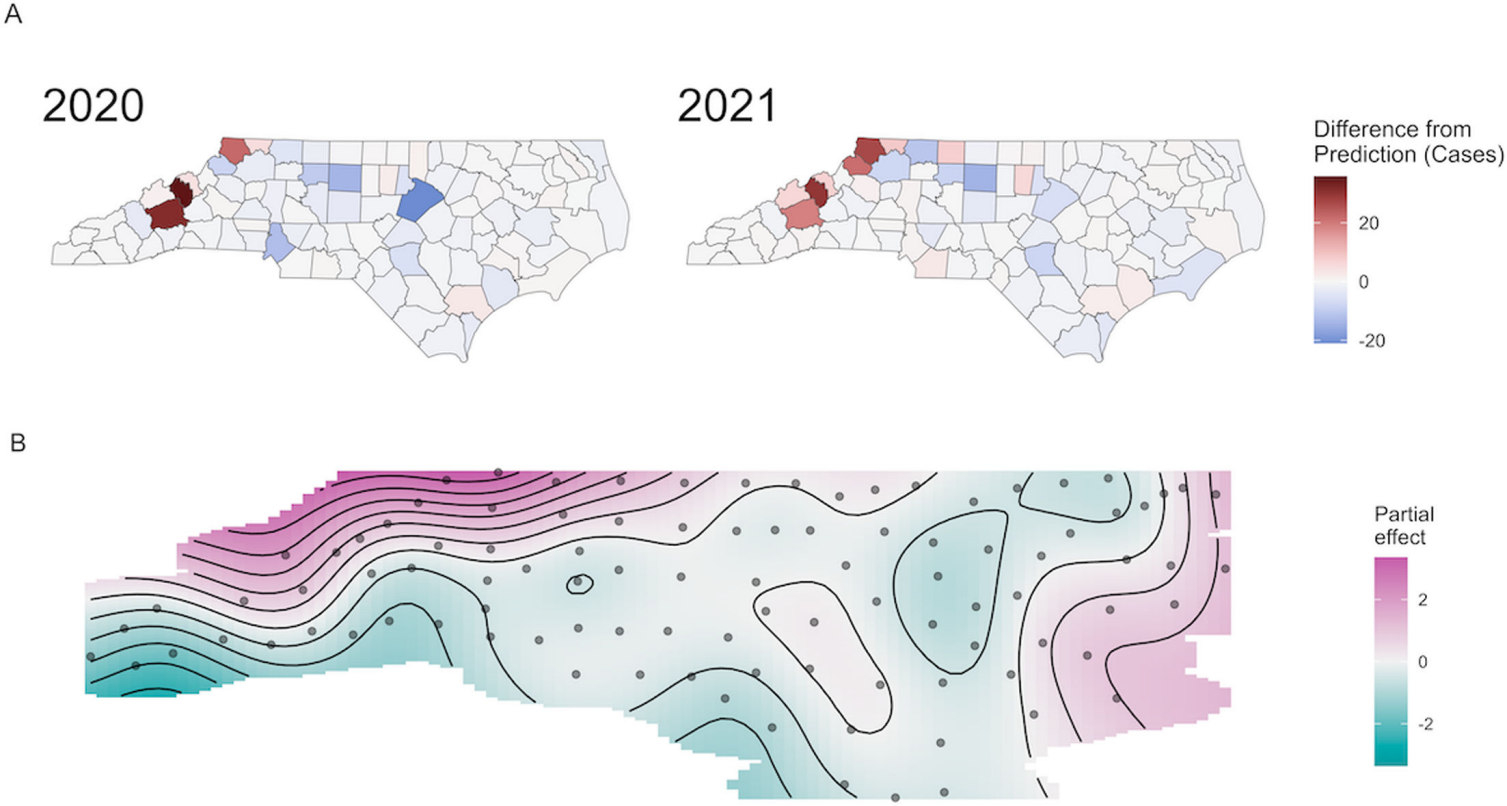
(A) The difference between the predicted and actual number of reported cases in North Carolina by county in 2020 and 2021. The color scale represents the absolute difference between the predicted number of cases and the actual number of cases of Lyme disease reported by the NCDHHS, with lighter values representing more cases than would be expected being reported based on the model estimates. (B) The contour plot of the spatial effect in the fitted model for reported Lyme disease cases by county in North Carolina. The color scale represents the partial effect of the space on the likelihood of reported Lyme disease cases, with lighter values representing higher likelihood. NCDHHS: North Carolina Department of Health and Human Services.

In 2020, the most populous counties in North Carolina, including Wake, Mecklenburg, and Guilford, showed the highest under-ascertainment of cases (Figure 2 and Table S2 in Multimedia Appendix 1), suggesting that individuals in higher-populated counties demonstrated behavioral changes, resulting in lower cases being reported to local health departments. During the COVID-19 pandemic, many outpatient medical offices switched to telehealth platforms to protect patients and staff, and hospitals became overwhelmed with increased numbers of patients. People in densely populated areas may have avoided medical centers to decrease their risk of exposure to COVID-19, and by not receiving medical care, a diagnosis for Lyme disease may have been missed. Globally, there was wider adoption of telehealth as a consequence of the pandemic [22]. However, a systematic review of the challenges of telehealth during the pandemic found that the inability to conduct a comprehensive physical examination was the top barrier to diagnosis during a telehealth encounter [23]. This could be further compounded by the delay or inability to receive laboratory diagnostics for Lyme disease during the pandemic period. Furthermore, an estimated 41% of adults in the United States avoided or delayed medical care during the COVID-19 pandemic by June 30, 2020 [24], further underlining the high likelihood of under-ascertainment we detected.

In 2020, we also found that the counties that had the highest number of reported cases compared to their predicted cases included Ashe, Yancey, and Buncombe counties (Figure 2). These counties, found in Western North Carolina, include many popular outdoor travel and recreational spots such as the Blue Ridge Parkway, Asheville, and Mount Mitchell. This suggests that behavioral changes, such as increased travel and utilization of outdoor recreational activities during the COVID-19 pandemic, could have resulted in more exposure to vectors, including ticks, and a higher-than-normal number of reported cases of Lyme disease.

### Repercussions of Missed Diagnoses

Untreated Lyme disease can pose complications for patients, with symptoms of late-stage Lyme disease including Lyme arthritis, most commonly in the knees; neurological complications; and carditis, which may be life-threatening [4,25]. Late-stage Lyme disease may occur months to years following the initial infection, which could pose significant health concerns in the coming years for patients who were never diagnosed and treated appropriately. One could hypothesize that the lack of treatment for Lyme disease during this period could result in a subsequent increase in chronic Lyme disease, which shares many symptoms with post–COVID-19 condition [26]; however, this is development unknown at this time.

### Limitations

There were several limitations, particularly that exposures to Lyme disease differ by region, both throughout North Carolina and the United States in general. Lyme disease surveillance and the available data are captured by county of residence, which may not represent where the exposure occurred. Lyme disease includes a broad spectrum of symptoms and presentations across the different stages, suggesting that some reported cases may be due to another cause. As described above, Lyme disease case reports only represent those individuals who seek health care and are subsequently diagnosed; thus, these numbers more generally represent an underestimate of the true burden of Lyme disease. Additionally, the case definition for the reporting of Lyme disease has been modified multiple times over the years, and the lack of good diagnostic tests impacts surveillance.

A key limitation to our study is the assumption of using historical reported Lyme disease cases prior to the pandemic period to generate the counterfactual estimate of Lyme disease cases in the absence of the pandemic. While this is a strong assumption, it serves to establish a likely lower bound for the number of under-ascertained Lyme disease cases due to variations in exposure and health care–seeking behaviors during the pandemic period. Furthermore, we were unable to conduct our analysis of month-to-month, estimated under-ascertainment rates at the national level due to a lack of available data. Other unrecorded and unobserved confounders are likely present in the recorded data. Our analysis could be improved by incorporating measures of greenspace utilization at appropriate spatial and temporal scales, both before and after the pandemic, to better refine the estimate for the likely number of cases given the changing exposure. Previous teaching emphasizing the lack of Lyme disease cases in North Carolina to minimize overdiagnosis and overtreatment is now superseded by the growth of this emerging infection.

### Conclusions

The COVID-19 pandemic had substantial effects on the passive surveillance of Lyme disease and likely ascertainment of cases. Public health officials should be wary of using data that have substantial variation in the ascertainment rates of cases to estimate secular trends and detect outbreaks. Future surveillance approaches that are less sensitive to wide variation in ascertainment rates should be developed.

## Supplementary material

10.2196/56571 Multimedia Appendix 1 Supplemental methods, 7-day rolling average value of population-weighted mobility to parks in North Carolina, fitted monthly smooth coefficients, 7-day incidence of COVID-19 cases in the United States, spatiotemporal regression model summary, and estimated ascertainment rates and reported cases of Lyme disease.

## Abbreviations

CDC: Centers for Disease Control and Prevention
NCDHHS: North Carolina Department of Health and Human Services

## References

[1] Wagner A (2022). How has the COVID-19 pandemic affected outdoor recreation in America?. Penn State University.

[2] Doubleday A, Choe Y, Busch Isaksen T, Miles S, Errett NA (2021). How did outdoor biking and walking change during covid-19?: a case study of three U.S. cities. PLoS One.

[3] Bristowe A, Heckert M (2023). How the COVID-19 pandemic changed patterns of green infrastructure use: a scoping review. Urban For Urban Green.

[4] Skar GL, Simonsen KA (2024). StatPearls.

[5] Stone BL, Tourand Y, Brissette CA (2017). Brave new worlds: the expanding universe of Lyme disease. Vector Borne Zoonotic Dis.

[6] McCormick DW, Kugeler KJ, Marx GE, Jayanthi P, Dietz S, Mead P (2021). Effects of COVID-19 pandemic on reported Lyme disease, United States, 2020. Emerg Infect Dis.

[7] Xu S, Glenn S, Sy L (2021). Impact of the COVID-19 pandemic on health care utilization in a large integrated health care system: retrospective cohort study. J Med Internet Res.

[8] Blumenthal D, Fowler EJ, Abrams M, Collins SR (2020). COVID-19 - implications for the health care system. N Engl J Med.

[9] Murray J, Cohen AL, Quah SR (2017). International Encyclopedia of Public Health.

[10] Unkel S, Farrington CP, Garthwaite PH, Robertson C, Andrews N (2012). Statistical methods for the prospective detection of infectious disease outbreaks: a review. J R Stat Soc Ser A Stat Soc.

[11] (2022). Lyme disease (Borrelia burgdorferi) 2022 case definition. Centers for Disease Control and Prevention.

[12] (2022). Lyme disease surveillance data. Centers for Disease Control and Prevention.

[13] (2023). NC DPH: communicable disease facts & figures. North Carolina Department of Health and Human Services.

[14] Population division. United Nations.

[15] (2023). County/state population projections. North Carolina Office of State Budget and Management.

[16] (2021). Lyme disease (Borrelia burgdorferi). Centers for Disease Control and Prevention.

[17] Bürkner PC (2017). brms: an R package for Bayesian multilevel models Using Stan. J Stat Softw.

[18] Vehtari A, Simpson D, Gelman A, Yao Y, Gabry J (2024). Pareto smoothed importance sampling. arXiv.

[19] Wood SN (2017). Generalized Additive Models: An Introduction with R.

[20] Eisen L, Eisen RJ (2023). Changes in the geographic distribution of the blacklegged tick, Ixodes scapularis, in the United States. Ticks Tick Borne Dis.

[21] Gibbons CL, Mangen MJJ, Plass D (2014). Measuring underreporting and under-ascertainment in infectious disease datasets: a comparison of methods. BMC Public Health.

[22] Omboni S, Padwal RS, Alessa T (2022). The worldwide impact of telemedicine during COVID-19: current evidence and recommendations for the future. Connect Health.

[23] Khoshrounejad F, Hamednia M, Mehrjerd A (2021). Telehealth-based services during the COVID-19 pandemic: a systematic review of features and challenges. Front Public Health.

[24] Czeisler MÉ, Lane RI, Orellana RC (2022). Perception of local COVID-19 transmission and use of preventive behaviors among adults with recent SARS-CoV-2 infection - Illinois and Michigan, June 1-July 31, 2022. MMWR Morb Mortal Wkly Rep.

[25] Forrester JD, Mead P (2014). Third-degree heart block associated with Lyme carditis: review of published cases. Clin Infect Dis.

[26] Soriano JB, Murthy S, Marshall JC, Relan P, Diaz JV, WHO Clinical Case Definition Working Group on Post-COVID-19 Condition (2022). A clinical case definition of post-COVID-19 condition by a Delphi consensus. Lancet Infect Dis.

[27] DeWitt ME (2024). Wf-id/lyme-ascertainment: publication code (v1.0.0). Zenodo.

