# Supplementary material for: Lyme Disease Under-Ascertainment During the COVID-19 Pandemic in the United States: Retrospective Study
Source: JMIR Public Health Surveill. 2024 Sep 12;10:e56571. doi: 10.2196/56571 (PMC11411844; doi:10.2196/56571)
Supplement: Multimedia Appendix 1 [file publichealth-v10-e56571-s001.docx]

# Multimedia Appendix 1

# Supplemental Methods

Cyclic cubic regression splines with 12 knots were utilized to account for the seasonal dynamics of infection during the year.

# Figures


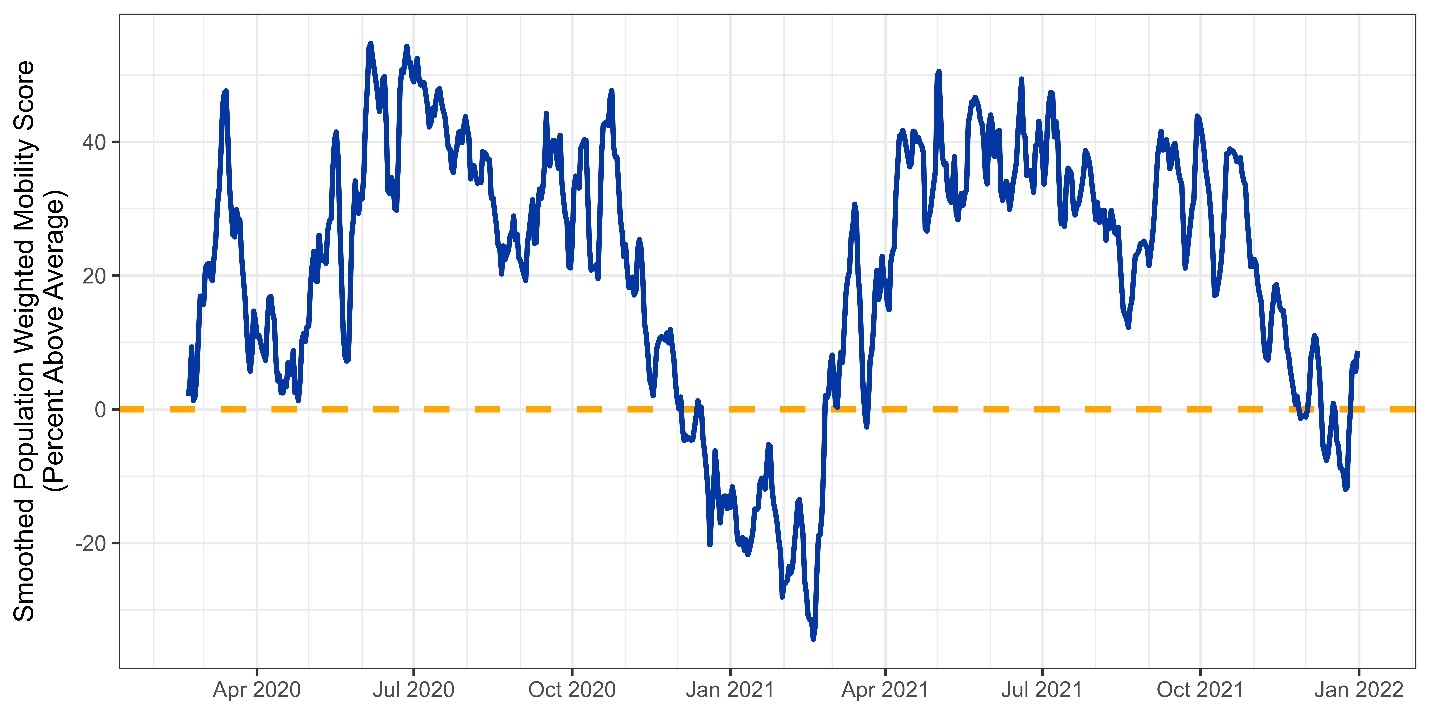


**Figure S1.** Seven-day rolling average value of population-weighted mobility to parks in North Carolina using Google COVID-19 Community Mobility Reports(1) compared to baseline values where values greater than zero indicate an increase in attendance. Population weights from the American Community Survey. Dashed, orange line represents zero (no change from baseline period).


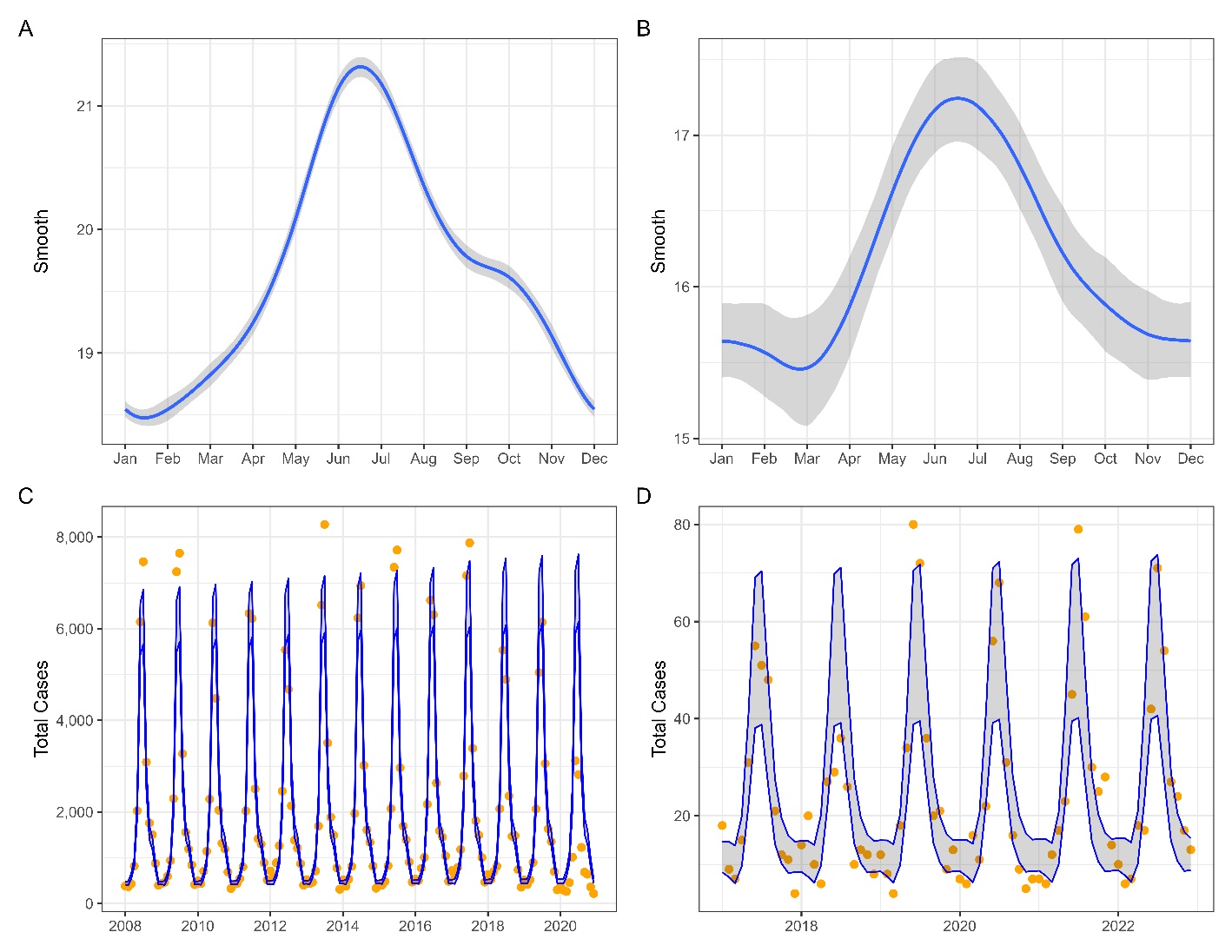


**Figure S2.** Fitted monthly smooth coefficients in the United States model (A) and North Carolina (B). The actual (points) and predicted number (line) of reported Lyme disease cases by month for the United States (C) and North Carolina (D). Note that 95% credible intervals are shown.


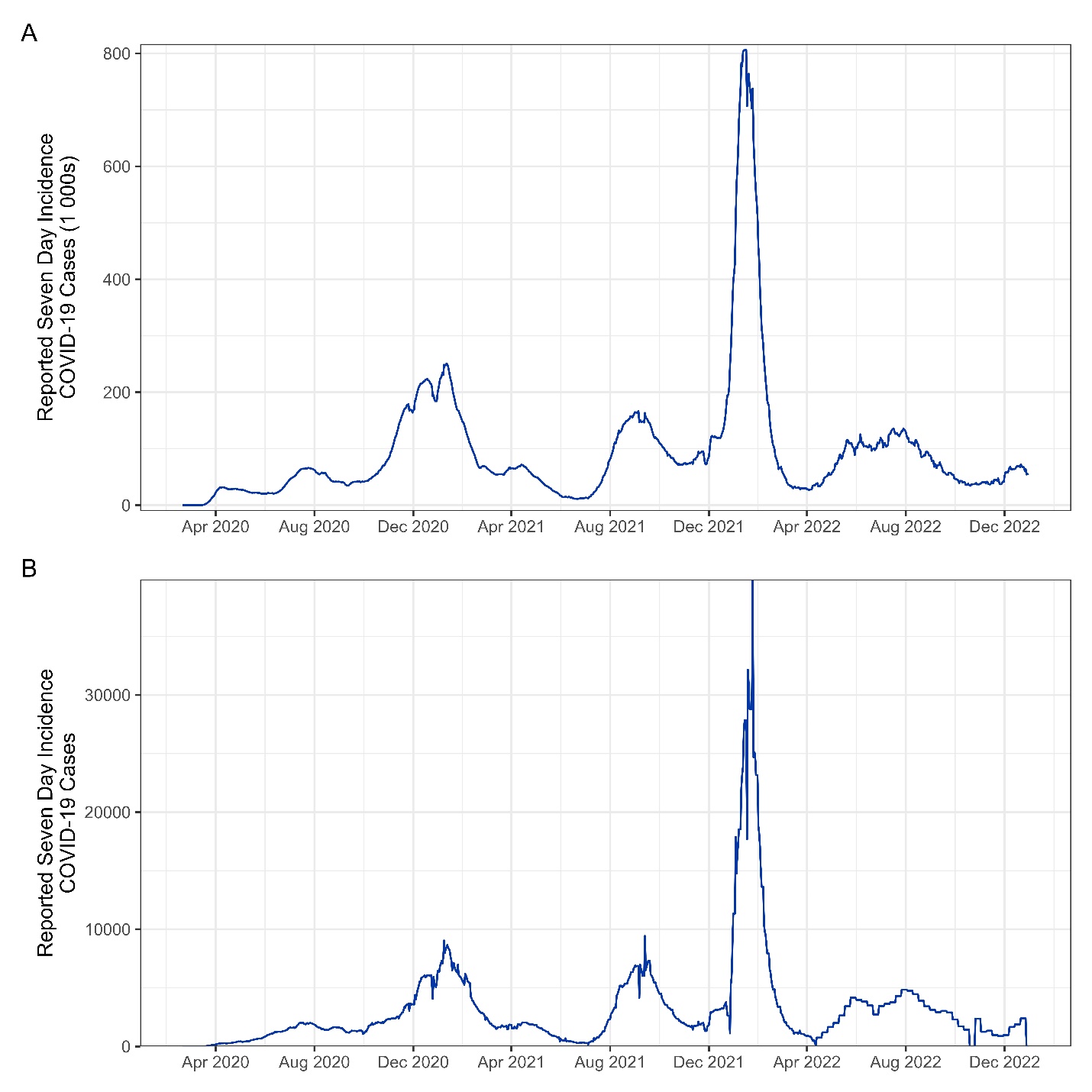


**Figure S3.** The reported seven day incidence of COVID-19 cases in the United States (A) and North Carolina (B) between March 1, 2020 and December 31, 2022. Data from the Johns Hopkins Covid-19 Dashboard(2) using the covidcast R package(3).

**Table S1.** Spatio-temporal regression model summary for the number of Lyme disease cases for North Carolina by county.

| **Characteristic** | **exp(Beta)** | **95% CI**^1^ | **p-value** |
| --- | --- | --- | --- |
| Year | 1.02 | 0.96, 1.09 | 0.4 |
| s(LngNBR,LatNBR) |  |  | <0.001 |
| ^1^CI = Confidence Interval | | | |

**Table S2.** Estimated ascertainment rates and reported cases of Lyme disease by North Carolina in 2020 and 2023.

|  | 2020 | | | 2021 | | |
| --- | --- | --- | --- | --- | --- | --- |
|  | Reported Case*^1^* | Estimated Cases (95% CI)*^2^* | Estimated Difference (95% CI)*^2^* | Reported Cases*^1^* | Estimated Cases (95% CI)*^2^* | Estimated Difference (95% CI)*^2^* |
| Alamance | 4 | 3.6 (2.2 to 5) | 0.4 (-1 to 1.8) | 3 | 3.8 (2.2 to 5.4) | -0.8 (-2.4 to 0.8) |
| Alexander | 1 | 1.2 (0.7 to 1.8) | -0.2 (-0.8 to 0.3) | 0 | 1.3 (0.7 to 1.8) | -1.3 (-1.8 to -0.7) |
| Alleghany | 12 | 7 (4.2 to 9.8) | 5 (2.2 to 7.8) | 16 | 7.3 (4.1 to 10.5) | 8.7 (5.5 to 11.9) |
| Anson | 1 | 0.4 (0.2 to 0.7) | 0.6 (0.3 to 0.8) | 0 | 0.4 (0.2 to 0.7) | -0.4 (-0.7 to -0.2) |
| Ashe | 36 | 13.7 (8 to 19.5) | 22.3 (16.5 to 28) | 41 | 14.1 (7.8 to 20.5) | 26.9 (20.5 to 33.2) |
| Avery | 1 | 1.2 (0.7 to 1.8) | -0.2 (-0.8 to 0.3) | 1 | 1.3 (0.7 to 1.9) | -0.3 (-0.9 to 0.3) |
| Beaufort | 0 | 2.2 (1 to 3.4) | -2.2 (-3.4 to -1) | 0 | 2.3 (1 to 3.6) | -2.3 (-3.6 to -1) |
| Bertie | 0 | 0.3 (0.1 to 0.5) | -0.3 (-0.5 to -0.1) | 1 | 0.3 (0.1 to 0.5) | 0.7 (0.5 to 0.9) |
| Bladen | 0 | 0.7 (0.3 to 1.1) | -0.7 (-1.1 to -0.3) | 0 | 0.7 (0.3 to 1.1) | -0.7 (-1.1 to -0.3) |
| Brunswick | 1 | 2.3 (1.1 to 3.6) | -1.3 (-2.6 to -0.1) | 0 | 2.5 (1.1 to 3.9) | -2.5 (-3.9 to -1.1) |
| Buncombe | 47 | 15.5 (9.4 to 21.5) | 31.5 (25.5 to 37.6) | 35 | 16 (9.2 to 22.7) | 19 (12.3 to 25.8) |
| Burke | 2 | 1.3 (0.7 to 1.9) | 0.7 (0.1 to 1.3) | 0 | 1.4 (0.7 to 2) | -1.4 (-2 to -0.7) |
| Cabarrus | 2 | 4.2 (2.7 to 5.8) | -2.2 (-3.8 to -0.7) | 1 | 4.4 (2.6 to 6.2) | -3.4 (-5.2 to -1.6) |
| Caldwell | 1 | 2.5 (1.5 to 3.5) | -1.5 (-2.5 to -0.5) | 4 | 2.6 (1.5 to 3.6) | 1.4 (0.4 to 2.5) |
| Camden | 0 | 0.4 (0.1 to 0.6) | -0.4 (-0.6 to -0.1) | 0 | 0.4 (0.1 to 0.7) | -0.4 (-0.7 to -0.1) |
| Carteret | 5 | 4.5 (1.7 to 7.3) | 0.5 (-2.3 to 3.3) | 0 | 4.6 (1.7 to 7.6) | -4.6 (-7.6 to -1.7) |
| Caswell | 1 | 0.6 (0.3 to 1) | 0.4 (0 to 0.7) | 1 | 0.6 (0.3 to 1) | 0.4 (0 to 0.7) |
| Catawba | 1 | 2.9 (1.7 to 4.1) | -1.9 (-3.1 to -0.7) | 3 | 3 (1.7 to 4.3) | 0 (-1.3 to 1.3) |
| Chatham | 1 | 1.8 (1 to 2.6) | -0.8 (-1.6 to 0) | 1 | 1.9 (1 to 2.8) | -0.9 (-1.8 to 0) |
| Cherokee | 0 | 0.2 (-0.1 to 0.5) | -0.2 (-0.5 to 0.1) | 0 | 0.2 (-0.1 to 0.5) | -0.2 (-0.5 to 0.1) |
| Chowan | 0 | 0.2 (0.1 to 0.3) | -0.2 (-0.3 to -0.1) | 0 | 0.2 (0.1 to 0.3) | -0.2 (-0.3 to -0.1) |
| Clay | 0 | 0 (0 to 0.1) | 0 (-0.1 to 0) | 0 | 0 (0 to 0.1) | 0 (-0.1 to 0) |
| Cleveland | 0 | 0.9 (0.4 to 1.4) | -0.9 (-1.4 to -0.4) | 1 | 1 (0.4 to 1.5) | 0 (-0.5 to 0.6) |
| Columbus | 0 | 0.8 (0.2 to 1.3) | -0.8 (-1.3 to -0.2) | 0 | 0.8 (0.2 to 1.4) | -0.8 (-1.4 to -0.2) |
| Craven | 4 | 3.7 (2 to 5.4) | 0.3 (-1.4 to 2) | 2 | 3.8 (2 to 5.6) | -1.8 (-3.6 to 0) |
| Cumberland | 3 | 8.6 (5 to 12.3) | -5.6 (-9.3 to -2) | 1 | 9 (4.9 to 13.1) | -8 (-12.1 to -3.9) |
| Currituck | 0 | 2.1 (0.7 to 3.5) | -2.1 (-3.5 to -0.7) | 1 | 2.2 (0.7 to 3.8) | -1.2 (-2.8 to 0.3) |
| Dare | 2 | 2.8 (0.9 to 4.8) | -0.8 (-2.8 to 1.1) | 2 | 2.9 (0.9 to 5) | -0.9 (-3 to 1.1) |
| Davidson | 0 | 2.9 (1.7 to 4.1) | -2.9 (-4.1 to -1.7) | 3 | 3 (1.7 to 4.3) | 0 (-1.3 to 1.3) |
| Davie | 0 | 0.8 (0.5 to 1.2) | -0.8 (-1.2 to -0.5) | 0 | 0.9 (0.5 to 1.2) | -0.9 (-1.2 to -0.5) |
| Duplin | 0 | 0.8 (0.4 to 1.2) | -0.8 (-1.2 to -0.4) | 0 | 0.8 (0.4 to 1.3) | -0.8 (-1.3 to -0.4) |
| Durham | 2 | 6.9 (4.3 to 9.5) | -4.9 (-7.5 to -2.3) | 2 | 7.2 (4.2 to 10.1) | -5.2 (-8.1 to -2.2) |
| Edgecombe | 1 | 0.7 (0.3 to 1) | 0.3 (0 to 0.7) | 1 | 0.7 (0.3 to 1) | 0.3 (0 to 0.7) |
| Forsyth | 3 | 13 (8.4 to 17.6) | -10 (-14.6 to -5.4) | 5 | 13.3 (8.1 to 18.5) | -8.3 (-13.5 to -3.1) |
| Franklin | 0 | 1.4 (0.8 to 2) | -1.4 (-2 to -0.8) | 0 | 1.5 (0.8 to 2.1) | -1.5 (-2.1 to -0.8) |
| Gaston | 2 | 3.5 (1.9 to 5.1) | -1.5 (-3.1 to 0.1) | 3 | 3.7 (1.8 to 5.5) | -0.7 (-2.5 to 1.2) |
| Gates | 0 | 0.1 (0 to 0.2) | -0.1 (-0.2 to 0) | 1 | 0.1 (0 to 0.3) | 0.9 (0.7 to 1) |
| Graham | 0 | 0.1 (0 to 0.2) | -0.1 (-0.2 to 0) | 0 | 0.1 (0 to 0.2) | -0.1 (-0.2 to 0) |
| Granville | 3 | 1.7 (0.9 to 2.5) | 1.3 (0.5 to 2.1) | 1 | 1.7 (0.9 to 2.6) | -0.7 (-1.6 to 0.1) |
| Greene | 0 | 0.2 (0.1 to 0.4) | -0.2 (-0.4 to -0.1) | 0 | 0.2 (0.1 to 0.4) | -0.2 (-0.4 to -0.1) |
| Guilford | 1 | 15.1 (9.5 to 20.6) | -14.1 (-19.6 to -8.5) | 1 | 15.5 (9.2 to 21.7) | -14.5 (-20.7 to -8.2) |
| Halifax | 1 | 0.8 (0.3 to 1.3) | 0.2 (-0.3 to 0.7) | 0 | 0.8 (0.3 to 1.3) | -0.8 (-1.3 to -0.3) |
| Harnett | 1 | 3.9 (2.3 to 5.4) | -2.9 (-4.4 to -1.3) | 2 | 4 (2.3 to 5.8) | -2 (-3.8 to -0.3) |
| Haywood | 0 | 3.1 (1.5 to 4.7) | -3.1 (-4.7 to -1.5) | 2 | 3.2 (1.4 to 4.9) | -1.2 (-2.9 to 0.6) |
| Henderson | 1 | 2.2 (1 to 3.4) | -1.2 (-2.4 to 0) | 3 | 2.3 (1 to 3.6) | 0.7 (-0.6 to 2) |
| Hertford | 0 | 0.3 (0 to 0.5) | -0.3 (-0.5 to 0) | 0 | 0.3 (0 to 0.5) | -0.3 (-0.5 to 0) |
| Hoke | 0 | 0.8 (0.4 to 1.2) | -0.8 (-1.2 to -0.4) | 0 | 0.9 (0.4 to 1.3) | -0.9 (-1.3 to -0.4) |
| Hyde | 0 | 0.2 (0.1 to 0.4) | -0.2 (-0.4 to -0.1) | 1 | 0.2 (0.1 to 0.4) | 0.8 (0.6 to 0.9) |
| Iredell | 2 | 3.8 (2.3 to 5.2) | -1.8 (-3.2 to -0.3) | 5 | 3.9 (2.3 to 5.6) | 1.1 (-0.6 to 2.7) |
| Jackson | 0 | 0.5 (0.1 to 0.9) | -0.5 (-0.9 to -0.1) | 1 | 0.5 (0.1 to 0.9) | 0.5 (0.1 to 0.9) |
| Johnston | 3 | 4 (2.3 to 5.7) | -1 (-2.7 to 0.7) | 4 | 4.3 (2.4 to 6.2) | -0.3 (-2.2 to 1.6) |
| Jones | 1 | 0.2 (0.1 to 0.3) | 0.8 (0.7 to 0.9) | 0 | 0.2 (0.1 to 0.3) | -0.2 (-0.3 to -0.1) |
| Lee | 1 | 1.6 (0.9 to 2.3) | -0.6 (-1.3 to 0.1) | 1 | 1.7 (0.9 to 2.5) | -0.7 (-1.5 to 0.1) |
| Lenoir | 0 | 0.8 (0.4 to 1.1) | -0.8 (-1.1 to -0.4) | 1 | 0.8 (0.4 to 1.2) | 0.2 (-0.2 to 0.6) |
| Lincoln | 2 | 1.3 (0.8 to 1.9) | 0.7 (0.1 to 1.2) | 2 | 1.4 (0.8 to 2) | 0.6 (0 to 1.2) |
| Macon | 0 | 0.2 (0 to 0.3) | -0.2 (-0.3 to 0) | 0 | 0.2 (0 to 0.3) | -0.2 (-0.3 to 0) |
| Madison | 5 | 3.5 (1.7 to 5.4) | 1.5 (-0.4 to 3.3) | 10 | 3.7 (1.7 to 5.6) | 6.3 (4.4 to 8.3) |
| Martin | 0 | 0.5 (0.2 to 0.7) | -0.5 (-0.7 to -0.2) | 0 | 0.5 (0.2 to 0.7) | -0.5 (-0.7 to -0.2) |
| McDowell | 0 | 0.8 (0.5 to 1.2) | -0.8 (-1.2 to -0.5) | 0 | 0.9 (0.4 to 1.3) | -0.9 (-1.3 to -0.4) |
| Mecklenburg | 9 | 21.4 (13.2 to 29.6) | -12.4 (-20.6 to -4.2) | 23 | 22 (12.9 to 31.1) | 1 (-8.1 to 10.1) |
| Mitchell | 5 | 0.9 (0.4 to 1.3) | 4.1 (3.7 to 4.6) | 8 | 0.9 (0.4 to 1.3) | 7.1 (6.7 to 7.6) |
| Montgomery | 0 | 0.6 (0.3 to 0.8) | -0.6 (-0.8 to -0.3) | 0 | 0.6 (0.3 to 0.9) | -0.6 (-0.9 to -0.3) |
| Moore | 2 | 2.1 (1.1 to 3) | -0.1 (-1 to 0.9) | 2 | 2.2 (1.1 to 3.2) | -0.2 (-1.2 to 0.9) |
| Nash | 0 | 1.2 (0.6 to 1.9) | -1.2 (-1.9 to -0.6) | 0 | 1.3 (0.6 to 1.9) | -1.3 (-1.9 to -0.6) |
| New Hanover | 1 | 3.9 (1.7 to 6) | -2.9 (-5 to -0.7) | 0 | 4 (1.7 to 6.4) | -4 (-6.4 to -1.7) |
| Northampton | 0 | 0.3 (0.1 to 0.5) | -0.3 (-0.5 to -0.1) | 0 | 0.3 (0.1 to 0.5) | -0.3 (-0.5 to -0.1) |
| Onslow | 2 | 5.6 (2.7 to 8.5) | -3.6 (-6.5 to -0.7) | 8 | 5.8 (2.7 to 9) | 2.2 (-1 to 5.3) |
| Orange | 5 | 2.9 (1.8 to 4.1) | 2.1 (0.9 to 3.2) | 9 | 3 (1.7 to 4.3) | 6 (4.7 to 7.3) |
| Pamlico | 0 | 0.8 (0.4 to 1.1) | -0.8 (-1.1 to -0.4) | 0 | 0.8 (0.4 to 1.1) | -0.8 (-1.1 to -0.4) |
| Pasquotank | 2 | 1 (0.4 to 1.6) | 1 (0.4 to 1.6) | 0 | 1 (0.4 to 1.7) | -1 (-1.7 to -0.4) |
| Pender | 4 | 1.2 (0.6 to 1.7) | 2.8 (2.3 to 3.4) | 3 | 1.3 (0.6 to 1.9) | 1.7 (1.1 to 2.4) |
| Perquimans | 0 | 0.2 (0.1 to 0.4) | -0.2 (-0.4 to -0.1) | 0 | 0.2 (0.1 to 0.4) | -0.2 (-0.4 to -0.1) |
| Person | 1 | 1 (0.5 to 1.5) | 0 (-0.5 to 0.5) | 2 | 1 (0.5 to 1.6) | 1 (0.4 to 1.5) |
| Pitt | 2 | 3.6 (1.9 to 5.3) | -1.6 (-3.3 to 0.1) | 2 | 3.7 (1.8 to 5.5) | -1.7 (-3.5 to 0.2) |
| Polk | 0 | 0.2 (0.1 to 0.3) | -0.2 (-0.3 to -0.1) | 0 | 0.2 (0.1 to 0.3) | -0.2 (-0.3 to -0.1) |
| Randolph | 0 | 3.3 (1.9 to 4.7) | -3.3 (-4.7 to -1.9) | 0 | 3.4 (1.9 to 5) | -3.4 (-5 to -1.9) |
| Richmond | 0 | 0.6 (0.3 to 1) | -0.6 (-1 to -0.3) | 0 | 0.6 (0.2 to 1) | -0.6 (-1 to -0.2) |
| Robeson | 0 | 1.2 (0.5 to 2) | -1.2 (-2 to -0.5) | 0 | 1.3 (0.5 to 2.1) | -1.3 (-2.1 to -0.5) |
| Rockingham | 4 | 4.5 (2.4 to 6.5) | -0.5 (-2.5 to 1.6) | 3 | 4.6 (2.4 to 6.9) | -1.6 (-3.9 to 0.6) |
| Rowan | 2 | 2.2 (1.3 to 3.1) | -0.2 (-1.1 to 0.7) | 1 | 2.2 (1.2 to 3.2) | -1.2 (-2.2 to -0.2) |
| Rutherford | 0 | 0.5 (0.2 to 0.9) | -0.5 (-0.9 to -0.2) | 0 | 0.5 (0.2 to 0.9) | -0.5 (-0.9 to -0.2) |
| Sampson | 1 | 1.5 (0.7 to 2.4) | -0.5 (-1.4 to 0.3) | 0 | 1.6 (0.7 to 2.5) | -1.6 (-2.5 to -0.7) |
| Scotland | 0 | 0.4 (0.1 to 0.6) | -0.4 (-0.6 to -0.1) | 1 | 0.4 (0.1 to 0.6) | 0.6 (0.4 to 0.9) |
| Stanly | 0 | 1.3 (0.7 to 1.8) | -1.3 (-1.8 to -0.7) | 1 | 1.3 (0.7 to 1.9) | -0.3 (-0.9 to 0.3) |
| Stokes | 2 | 4.2 (2.5 to 6) | -2.2 (-4 to -0.5) | 12 | 4.4 (2.4 to 6.4) | 7.6 (5.6 to 9.6) |
| Surry | 10 | 14.6 (8.6 to 20.6) | -4.6 (-10.6 to 1.4) | 4 | 15 (8.4 to 21.6) | -11 (-17.6 to -4.4) |
| Swain | 0 | 0.2 (0 to 0.5) | -0.2 (-0.5 to 0) | 0 | 0.2 (0 to 0.5) | -0.2 (-0.5 to 0) |
| Transylvania | 0 | 0.5 (0.2 to 0.8) | -0.5 (-0.8 to -0.2) | 1 | 0.5 (0.2 to 0.9) | 0.5 (0.1 to 0.8) |
| Tyrrell | 0 | 0.1 (0 to 0.2) | -0.1 (-0.2 to 0) | 0 | 0.1 (0 to 0.2) | -0.1 (-0.2 to 0) |
| Union | 5 | 5 (2.6 to 7.4) | 0 (-2.4 to 2.4) | 8 | 5.2 (2.6 to 7.9) | 2.8 (0.1 to 5.4) |
| Vance | 0 | 1.3 (0.6 to 2) | -1.3 (-2 to -0.6) | 1 | 1.3 (0.6 to 2) | -0.3 (-1 to 0.4) |
| Wake | 4 | 25 (15.1 to 34.9) | -21 (-30.9 to -11.1) | 20 | 26 (14.8 to 37.1) | -6 (-17.1 to 5.2) |
| Warren | 0 | 0.5 (0.2 to 0.8) | -0.5 (-0.8 to -0.2) | 0 | 0.5 (0.2 to 0.8) | -0.5 (-0.8 to -0.2) |
| Washington | 1 | 0.3 (0.1 to 0.5) | 0.7 (0.5 to 0.9) | 0 | 0.3 (0.1 to 0.5) | -0.3 (-0.5 to -0.1) |
| Watauga | 1 | 9.2 (5.6 to 12.9) | -8.2 (-11.9 to -4.6) | 31 | 9.4 (5.4 to 13.5) | 21.6 (17.5 to 25.6) |
| Wayne | 0 | 1.4 (0.7 to 2) | -1.4 (-2 to -0.7) | 2 | 1.4 (0.7 to 2.1) | 0.6 (-0.1 to 1.3) |
| Wilkes | 8 | 10.5 (6.8 to 14.2) | -2.5 (-6.2 to 1.2) | 3 | 10.7 (6.6 to 14.9) | -7.7 (-11.9 to -3.6) |
| Wilson | 1 | 0.8 (0.4 to 1.3) | 0.2 (-0.3 to 0.6) | 1 | 0.8 (0.4 to 1.3) | 0.2 (-0.3 to 0.6) |
| Yadkin | 1 | 2.2 (1.4 to 3) | -1.2 (-2 to -0.4) | 2 | 2.2 (1.3 to 3.1) | -0.2 (-1.1 to 0.7) |
| Yancey | 37 | 1.2 (0.7 to 1.7) | 35.8 (35.3 to 36.3) | 31 | 1.2 (0.6 to 1.8) | 29.8 (29.2 to 30.4) |
| *^1^*Lyme disease cases reported to the North Carolina Department of Health and Human Services | | | | | | |
| *^2^*CI, 95% Confidence Interval | | | | | | |

# Data

Code and data supporting this analysis are available at https://github.com/wf-id/lyme-ascertainment.git.

# References

1. Google. COVID-19 Community Mobility Report. 2022 [cited 2023 Nov 3]. COVID-19 Community Mobility Report. Available from: https://www.google.com/covid19/mobility?hl=en

2. Johns Hopkins Coronavirus Resource Center [Internet]. [cited 2023 Nov 3]. COVID-19 Map. Available from: https://coronavirus.jhu.edu/map.html

3. Reinhart A, Brooks L, Jahja M, Rumack A, Tang J, Agrawal S, et al. An open repository of real-time COVID-19 indicators. Proceedings of the National Academy of Sciences [Internet]. 2021 Dec 21 [cited 2023 Nov 3];118(51):e2111452118. Available from: https://www.pnas.org/doi/10.1073/pnas.2111452118
